# Reply to Cantarelli et al. Chronic Recurrent Multifocal Osteomyelitis Associated with Crohn Disease: A Potential Role of Exclusion Diet? Comment on “Starz et al. The Modification of the Gut Microbiota via Selected Specific Diets in Patients with Crohn’s Disease. *Nutrients* 2021, *13*, 2125”

**DOI:** 10.3390/nu13114007

**Published:** 2021-11-10

**Authors:** Eliza Starz, Karolina Wzorek, Marcin Folwarski, Karolina Kaźmierczak-Siedlecka, Laura Stachowska, Katarzyna Przewłócka, Ewa Stachowska, Karolina Skonieczna-Żydecka

**Affiliations:** 1 Students’ Scientific Circle of Clinical Nutrition, Medical University of Gdańsk, 80-211 Gdańsk, Poland; eliza.starz@gumed.edu.pl (E.S.); wzorekka@gumed.edu.pl (K.W.); 2 Department of Clinical Nutrition and Dietetics, Medical University of Gdańsk, 80-211 Gdańsk, Poland; 3 Department of Surgical Oncology, Medical University of Gdańsk, 80-210 Gdansk, Poland; leokadia@gumed.edu.pl; 4 Department of Biochemical Sciences, Pomeranian Medical University in Szczecin, 71-460 Szczecin, Poland; laura.stachowska@pum.edu.pl (L.S.); karzyd@pum.edu.pl (K.S.-Ż.); 5 Department of Bioenergetics and Physiology of Exercise, Medical University of Gdańsk, 80-210 Gdańsk, Poland; kprzewlocka@gumed.edu.pl; 6 Department of Human Nutrition and Metabolomics, Pomeranian Medical University in Szczecin, 71-460 Szczecin, Poland; ewa.stachowska@pum.edu.pl

We congratulate Erika Cantarelli and colleagues for the presented case report in the comment entitled “Chronic Recurrent Multifocal Osteomyelitis associated to Crohn Disease (CD): a potential role of exclusion diet?”. Thank you for reporting your thoughts regarding our recently published review paper “The Modification of the Gut Microbiota via Selected Specific Diets in Patients with Crohn’s Disease” in *Nutrients* [1]. We briefly presented dysbiotic alterations of gut microbiota in Crohn’s disease and characterized several types of diets (such as Crohn’s disease exclusion diet (CDED), low-FODMAP, gluten-free, lactose-free, ketogenic, specific carbohydrate, and Paleo diet) in the treatment of CD regarding their impact on gut microbiota. 

In the comment, Cantarelli and colleagues observed clinical improvement after the introduction of the aforementioned CDED in combination with infliximab and methotrexate in a pediatric patient with CD and chronic recurrent multifocal osteomyelitis (CRMO). Recently, Scarallo et al. have presented a case series regarding CDED in children with CD. The authors concluded that this diet in combination with partial enteral nutrition (PEN) is effective in children with mild to moderate CD [2]. Other authors reported that CDED induces both rapid response and remission in children suffering from active Crohn’s disease [3]. 

We agree that little is known on the effect of diet interventions on extraintestinal manifestations of CD or CRMO. Studies showing interleukin-1β (IL-1β) as pivotal players in the development of CRMO and Crohn’s disease do exist [4,5]. In the experimental study, a diet rich in high saturated fats and cholesterol (HFD) protected the mice from osteomyelitis. The authors pointed that diet-linked alterations in the gut microbiota might have been responsible for a specific, favorable, immunological effect. HFD diet resulted in a decreased abundance of *Provotella* and increased abundance of *Lactobacillus* species, both found to be associated with a diminished IL-1β synthesis [4]. The CDED diet in combination with partial enteral nutrition (PEN) has been shown to affect microbiota diversity. The use of this dietary approach resulted in a lower counts of many pathogenic microorganisms, including *Provotella* sp. On the other hand, the CDED + PEN diet caused an increase in *Firmicutes* bacteria, including *Lactobacillus* species [6]. Consequently, such a dietary approach might possess efficacy in CRMO treatment as presented by Cantarelli and colleagues.

Some new studies registered in ClinicalTrials.gov are designed to give additional knowledge in this area. In the trial no. NCT02341248, in children with CD subjected to Exclusive Enteral Nutrition (EEN), bacterial metabolism, composition in the gut and mucosal tissue are to be assessed together with inflammatory markers and dietary information. Another study has been including pediatric patients with CD (no. NCT02201693) and comparing cyclic exclusive EN for 2 weeks following 6 weeks of the diet with a repeated cycle every 8 weeks vs. EN (25% of caloric intake). The outcomes to be released include clinical and endoscopic remission, transmural healing, quality of life and growth pattern. In the NCT02843100 trial, which is still recruiting children with active mild or severe CD, CDED will be compared with modified Exclusive EN and PEN to assess clinical remission, microbiome composition, calprotectin, remission induction and mucosal healing. The dietary treatment has been recently acknowledged as add-on intervention in the course if gut related entities, and more studies are warranted, including our team.

Once again, we congratulate the authors of the comment for the successful treatment of CRMO and hope that future studies will provide more data on this topic. 
